# Breastfeeding among Latino Families in an Urban Pediatric Office Setting

**DOI:** 10.1155/2016/9278401

**Published:** 2016-11-17

**Authors:** Elizabeth Sloand, Chakra Budhathoki, Julia Junn, Dolly Vo, Victoria Lowe, Amy Pennington

**Affiliations:** ^1^Johns Hopkins University School of Nursing, 525 North Wolfe Street, Baltimore, MD 21205, USA; ^2^School of Nursing, The Catholic University of America, 620 Michigan Ave NE, Washington, DC 20064, USA

## Abstract

*Objective*. To determine the breastfeeding rate of Latino infants at an urban pediatric clinic in the first six months of life and to identify factors associated with breastfeeding.* Methods*. Investigators conducted a retrospective chart review of infants seen at the clinic in 2014 as part of a mixed methods study. Topics reviewed included demographics, infant health data, and feeding methods at 5 points in time. Bivariate correlations and cross-tabulations explored associations between variables.* Results*. Most of the mothers (75%) fed their newborns with both breastfeeding and formula (*las dos*). At 6 months, a majority were formula-fed only (55.9%). Approximately 10% of mothers exclusively breastfed their newborns, and the trend of exclusive breastfeeding remained steady through the 6-month visit. Over time, the number of mothers who exclusively bottle-feed their infants steadily rises. There were no statistical differences among the feeding method groups with regard to birth order of child, number of adults or children in the household, vaccination rate, number of sick visits, or infants' growth.* Conclusions*. More targeted attention to this population and other immigrant populations with culturally tailored interventions spanning the prenatal to early infancy periods could increase exclusive breastfeeding and ultimately improve child health.

## 1. Introduction

Although many health entities such as the World Health Organization (WHO) [1] and American Congress of Obstetricians and Gynecologists (ACOG) [2] recommend exclusive breastfeeding for the first 6 months of a child's life for optimal growth, development, and health, exclusive breastfeeding rates in the United States still fall short of national goals set by* Healthy People 2020* [3]. In 2014, exclusive breastfeeding rates were 41% at 3 months and only 19% at 7 months [4]. According to ACOG, the maternal and infant health benefits of breastfeeding are especially important to underserved women, who are disproportionately more likely to experience adverse health outcomes such as diabetes, obesity, and cardiovascular disease, all of which may improve with breastfeeding [2]. Hispanic children in particular have persistently had higher obesity prevalence rates (14.8%) compared to black infants (8.7%) and white infants (8.4%) [5]. Verstraete et al. [6] found that infants of recently immigrated Latina women who were breastfed up to 1 year of age had a decreased risk of obesity at 3 years of age. Additionally, a study conducted in Brazil found that breastfeeding duration positively impacts intelligence quotient (IQ), years of schooling, and income at 30 years of age [7]. With the multifaceted benefits of breastfeeding, many underserved communities have much to gain from healthcare efforts to improve exclusive and longer breastfeeding practices.

Though Latina mothers in the US have higher rates of breastfeeding* initiation* than the national average, only 18% breastfeed their infants* exclusively* [8]. Latina mothers are more likely to initiate breastfeeding but less likely to practice exclusive breastfeeding compared to other ethnic groups [5]. The practice of feeding infants both breastmilk and formula is a prevalent and culturally embedded practice among many Latina women and is referred to as* las dos cosas* [8]. Latina mothers are more likely than other groups to supplement formula feeding in the first 2 days of life [9]. By 5 months of age, 86% of infants were supplemented with formula.

In recent years, significant progress has been accomplished in informing and supporting mothers in exclusive breastfeeding within the hospital setting. However, more information is needed in order to provide interventions to improve breastfeeding duration and exclusivity in the community setting. By understanding the factors associated with increased breastfeeding rates outside the hospital setting, as well as the barriers to exclusive breastfeeding after discharge, providers can better assist mothers to exclusively breastfeed their infants through the first 6 months of life. The aims of this study were (1) to determine the method and rates of breastfeeding among Latina mothers and infants in the first 6 months of life and (2) to identify the demographic and social indicators of breastfeeding practices among Latina women. Our goal is to gain a better understanding of breastfeeding behaviors in this population so that we can ultimately improve policies and strategies that support optimal breastfeeding among mothers and infants in this clinic.

The World Health Organization [1] recommends that mothers practice exclusive breastfeeding for the first six months of life of an infant's life. Breastfeeding is considered the Gold Standard method of infant feeding and provides multiple benefits for mothers and infants. Maternal benefits of breastfeeding are well known to be both short and long term and include reduced stress in the postpartum period, reduced risk of breast and ovarian cancer, and reduced risk of cardiovascular problems and type 2 diabetes [10]. Birth spacing is also improved by breastfeeding, as increased duration and frequency of breastfeeding were associated with longer periods of amenorrhea [7].

Infant benefits from breastfeeding are numerous. Exclusively breastfeeding for six months insures that the infant achieves optimal growth, development, and health. According to a meta-analysis, which looked at children in low, middle, and high income countries, the significant benefits for children were evident regardless of income level [7]. In a United States study, Bartick and Reyes modeled costs of suboptimal breastfeeding and estimated 911 deaths annually could be prevented by optimal breastfeeding [11]. In terms of morbidity, Victora and colleagues' meta-analysis shows that worldwide about half of all diarrheal episodes (along with 72% of hospital admissions for diarrhea) and a third of respiratory infections (along with 57% hospital admissions for respiratory infections) could be avoided by breastfeeding [7]. Breastfeeding is also associated with a 9% reduction in asthma and a 68% reduction in dental malocclusion. Verstraete and colleagues [6] found that, for recently immigrated Latina mothers, breastfeeding for a year or more is associated with a healthier infant weight, specifically a decreased weight percentile for age, body mass index percentile and* z*-score for age, and waist circumference below the 90th percentile. Results also showed that breastfeeding for a year or longer was associated with a decreased risk of obesity and persisted after controlling for maternal BMI, marital status, education, country of origin, age, years of living in the United States, and child's birthweight.

Though breastfeeding proves beneficial for both mother and infant, numerous barriers exist, especially in the Latin/Hispanic population. One of the major barriers is the need to return to work. According to Besore [12], women in low-wage positions are unable to afford extending unpaid maternity leave or may not have the option to obtain a flexible schedule. Even when offered work accommodations, some women do not take advantage of them out of fear of embarrassment or being fired. Another barrier to breastfeeding is concern of having inadequate milk supply, which ties in with the belief that, in Latin culture, a heavier baby (*gordito*) is a healthier baby [5]. Acculturation also plays a pivotal role in hindering exclusive breastfeeding practices. According to Besore [12], the longer Latina immigrants live in the United States, the more they come to believe that the American way for infant feeding is through formula. Culturally, it is believed that breastfeeding is a practice of the poor, because of one's inability to purchase formula [13]. All these factors may contribute to the* las dos* feeding method. According to Waldrop [8], frequent reasons for* las dos* behaviors included the following: the baby being not satisfied because of insufficient breast milk, feeding breast milk along with formula providing the best nutrition for the baby, and the mother's intention of working outside the home while still feeding her child or the inconvenience of breastfeeding when the mother must be away.

## 2. Methods

### 2.1. Study Design

This study is part of a sequential mixed methods study completed in two phases. In phase 1, investigators sought to determine the rate of breastfeeding in the first six months of life among Latino infants seen at an urban health center and to identify any associated factors related to breastfeeding in this group. In phase 2, focus groups were conducted to explore mothers' perceptions regarding breastfeeding using qualitative methodology.

We report here on phase 1 of the study, the retrospective chart review using electronic medical records. Preliminary results of phase 1 were reported in a presentation in Atlanta, USA, in March 2016 [13].

### 2.2. Setting and Sample

The setting for this study was an urban hospital pediatric outpatient clinic in the northeast United States. The population included all children who are seen at the urban pediatric clinic for their health supervision visits. Inclusion criteria for the sample included those who were born in 2014 and had been a patient at the clinic for at least 6 months, whose mother identified as Hispanic or Latina, and whose mother was at least 18 years old. Babies who did not live with their mothers were excluded from the study. The sample consisted of 202 babies who fit the inclusion criteria of the study.

### 2.3. Procedure for Data Collection

The study protocol was approved by the Johns Hopkins Medicine Institutional Review Board and the Hopkins Bayview Medical Center Children's Medical Practice Research Review Committee. After ethics approval was granted by both the university and the clinic administration, research staff were trained to review and extract data from each patient record. Data collected included gender, race/ethnicity, country of origin, insurance coverage, number of children in the household, total number of children of the mother, birth order of infant, weight-for-length percentile, marital status of parents, number of episodic/sick visits and hospitalizations in the first 6 months of life, the infant's vaccination status in first 6 months of life, and the method of feeding at the newborn, 1-, 2-, 4-, and 6-month preventive health visits (breast milk or formula). All data was reviewed, cleaned, and monitored for accuracy.

### 2.4. Statistical Analysis

Following data entry, descriptive statistics were calculated to describe and summarize the data, for example, mean (SD) for continuous variables and* n* (%) for categorical variables. Bivariate correlations and cross-tabulations were performed to explore the associations between variables. The analysis was conducted using IBM SPSS Statistics software (IBM Corp., Armonk, NY). Friedman test was used due to the violation of one-way ANOVA. The study alpha level was set at *p* = 0.05.

## 3. Results

The average age of the mothers was 27 years with 35% of them being young (18–24 years) and over 50% being 25–35 years old. With respect to birth order, there was an even distribution between first, second, and third born. The majority of mothers (67%) were married or living with the father of the baby. Household size, number of adults, and children in the home varied widely. See Table 1 for detailed demographic information of the sample. The majority of the families were from four countries of origin: Honduras, Mexico, El Salvador, and Guatemala; half of the EMRs did not have country of origin information available.

**Figure 1:**
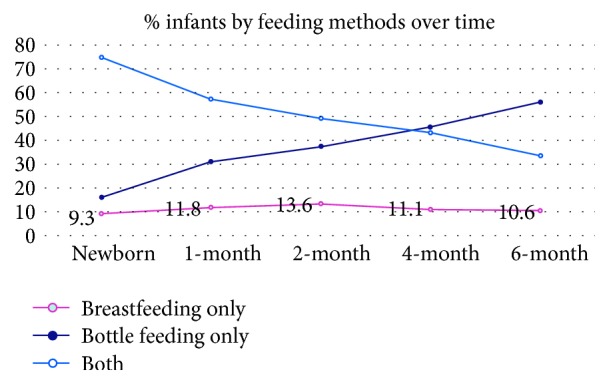
Feeding method in first 6 months of life.

Most of the mothers (75%) fed their newborns with both breastfeeding and formula feeding in the first week of life (*las dos*). At the 6-month visit, a majority of infants were formula fed only (55.9%); 33.5% were fed with both breast and formula. Approximately 10% of the mothers exclusively breastfed at the newborn visit and the trend of exclusive breastfeeding remained steady through to the 6-month visit. Over time, the number of mothers who exclusively bottle-fed their infants steadily rose, and correspondingly, the number who both breastfeed and bottle-feed steadily decreased (See Figure 1). There were no statistically significant differences between the feeding method groups (breastfeeding only, formula feeding only, and* las dos*) with regard to birth order of child, or the number of adults or children living in the household. The vaccination rate was not statistically different across three feeding method groups (*p* = 0.161) using Fisher's exact test. The difference in median number of sick visits across the three groups was not statistically significant (*p* = 0.216) using a Kruskal-Wallis test. There was no significant difference (*p* = 0.319) in infants' growth (weight for length) at the 6-month visit time between the feeding groups by one-way ANOVA.

## 4. Discussion

Since* las dos* mothers represent the majority of the population of mothers at the study clinic, the data for all mothers and* las dos* mothers are very similar. Consistent with the findings of Waldrop [8], Latina mothers at this urban clinic have higher rates of* any* breastfeeding initiation than the national and state average, even surpassing* Healthy People 2020* goals. However, the 6-month rate of any breastfeeding in this sample is 41.5%, which is significantly lower than both the* Healthy People 2020* goal of 60.6%, as well as national (49.4%), and Maryland state (60.1%) rate for any breastfeeding at 6 months [14]. In addition, exclusive breastfeeding rates in this study are much lower than* Healthy People 2020* goals, the national average, and the state average—which is also consistent with the findings in the Waldrop study of Latina mothers [8].

Of note, our finding that there was no significant different in infants' growth (weight for length) at the 6-month visit among the three feeding groups is intriguing. It is possible that differences in growth become apparent in the second half of the first year and so would not be seen in this study of feeding patterns in the first six months of life. Additionally, data about the variable “feeding method” was collected retrospectively from notation about feeding in the baby's EMR during routine well child visits. The retrospective nature of the data collection for this variable did not allow researchers to explore with the caregiver about the extent of breastfeeding, or control for differences in practitioners' classification of infant feeding type based on caregiver report. A mother who reports she is breastfeeding her infant might actually practice a breastfeeding pattern that would be described as “token” (less than 15 minutes per day), based on schema and framework for breastfeeding definition described by Labbok and Krasovec [15]. With “token” breastfeeding, a baby would be expected to demonstrate growth patterns consistent with formula feeding babies despite mother's report of breastfeeding [15].

This study shows a clear relationship between formula use in the early postpartum period and premature cessation of breastfeeding. The rate of exclusive breastmilk feeding remained relatively steady at around 10%, while many mothers who began feeding both breastmilk and formula quickly transitioned to formula-only feeding. Comparison of our results to the literature regarding the impact of formula supplementation is complicated by inconsistencies in the evidence. Kim et al. [16] identified prenatal intention to feed both formula and breastmilk to negatively impact duration of breastfeeding; however, the authors note recall bias may have affected the reliability of their findings. Furthermore, the study population did not include Latina mothers, and so results may not be transferrable to the Latina population. Holmes et al. [17] demonstrated shorter duration of breastfeeding for infants who were fed a combination of breastmilk and formula; however these findings were not noted in the Hispanic population. Their study also relied on recall of feeding methods for children under 6 years of age, with similar possibility for recall bias.

Given the potential negative impact of formula supplementation on breastfeeding duration demonstrated in this study, the phenomena of* las dos* as a feeding preference for Latina women may not be a benign cultural practice, but rather a public health threat. Careful exploration of the reasons for this practice can help in identification of strategies for promotion of exclusive breastfeeding in the Latina population. Formula supplementation practices among Latina mothers have been attributed to many factors, including unrealistic expectations about infant behavior, mothers' need for rest, inadequate knowledge about breastfeeding physiology and processes, and a perception that formula is a solution for breastfeeding problems [18]. Further factors include a lack of awareness of negative consequences of supplementation and a lack of awareness of medical recommendations for exclusive breastfeeding [11] as well as challenges faced by immigrant mothers that are related to acculturation [12].

## 5. Limitations

This study utilized EMR data that was sometimes difficult to locate in each record. For example, there was not one place in each record where the number of adults in the household was reliably documented. For some of the independent variables, the EMR was missing information. In some cases, the number of records with missing information was significant. For example, approximately half of the babies' charts did not include the country of origin for their families. In addition, we were unable to allow for multiple classifications of degree of breastfeeding due to the retrospective nature of the study. Finally, this study was performed in an urban pediatric office setting so the results are not generalizable to other populations. The results, however, can be used to inform others who are interested in optimizing breastfeeding promotional activities with Latina mothers and their infants.

## 6. Nursing Implications 

The WHO global strategy for infant nutrition and feeding includes a target goal for rates of exclusive breastmilk feeding at 50% [19]. Findings from this study suggest that those mothers who begin with exclusive breastmilk feeding continue with exclusive breastmilk feeding through 6 months; therefore efforts among maternal child nurses to promote, protect, and support exclusive breastfeeding may influence duration of breastfeeding. High formula supplementation rates in the hospital point towards a need for interventions across the continuum of care from the prenatal period, during the hospital stay, and continuing into the postpartum period to reduce formula use. Prenatally, better education is needed about the medical recommendations for infant feeding and anticipatory guidance about options for mothers experiencing difficulty in breastfeeding without resorting to formula. In addition to education about optimal feeding practices, there is a need to address culturally held beliefs about infant health and well-being, especially the idea that heavy babies are healthier.

Given the impact of acculturation on breastfeeding practices [12], the unique challenges faced by immigrant mothers must be addressed. With increased mobility and movement of populations across national borders, it is essential for nurses to develop the cultural awareness necessary to adeptly address the needs of populations such as immigrant mothers who reflect blended cultural influences. Implementation of culturally sensitive practices to support exclusive breastfeeding and help mothers to cope with challenges such as exhaustion and fussy infants would be expected to help a mother navigate the early and sometimes difficult days of parenting without resorting to formula as a solution to breastfeeding challenges.

## 7. Conclusion

The significantly low rate of exclusive breastfeeding at the initial newborn visit highlights the need to address upstream breastfeeding practices and policies, even before the first visit to the pediatric outpatient clinic. Perinatal nurses, lactation consultants, and obstetric health providers play critical roles in establishing optimal breastfeeding practices in the immediate postpartum period. Specifically, the negative correlation between the rate of any breastfeeding and formula-only feeding over time underscores the need to focus on promotion of exclusive breastmilk feeding when planning interventions to promote optimal practices among Latina mothers. Further study of this population, including qualitative exploration, will help guide health care providers to identify barriers and revise practices that enhance breastfeeding among Latina mothers. This could include clinic-specific education and anticipatory guidance. Increasing exclusive breastfeeding rates in all populations is a worthy worldwide goal given its important health benefits for both mothers and infants.

## Figures and Tables

**Table 1:** Demographics of sample.

*Average age of mothers (years ± SD)*	27.25 ± 5.82
*Age group*	
16–24 years old	70 (35%)
25–35 years old	109 (54.5%)
>35 years old	16 (8%)
Unknown	5 (2.5%)
Total	200 (100%)
*Birth order*	
1	57 (28.5%)
2	72 (36%)
3 or higher	69 (34.5%)
Unknown	2 (1%)
Total	200 (100%)
*Household information*	
*Number of children living in household*	
1	48 (24%)
2	57 (28.5%)
3 or more	60 (30%)
Unknown	35 (17.5%)
Total	200 (100%)
*Number of adults living in household*	
1	8 (4%)
2	81 (40.5%)
3 or more	79 (39.5%)
Unknown	32 (16%)
Total	200 (100%)
*Household type*	
Married	44 (22%)
Living together	90 (45%)
Separated	27 (13.5%)
Other	11 (5.5%)
Unknown	28 (14%)
Total	200 (100%)
